# Optimal sampling designs for estimation of *Plasmodium falciparum* clearance rates in patients treated with artemisinin derivatives

**DOI:** 10.1186/1475-2875-12-411

**Published:** 2013-11-13

**Authors:** Jennifer A Flegg, Philippe J Guérin, Francois Nosten, Elizabeth A Ashley, Aung Pyae Phyo, Arjen M Dondorp, Rick M Fairhurst, Duong Socheat, Steffen Borrmann, Anders Björkman, Andreas Mårtensson, Mayfong Mayxay, Paul N Newton, Delia Bethell, Youry Se, Harald Noedl, Mahamadou Diakite, Abdoulaye A Djimde, Tran T Hien, Nicholas J White, Kasia Stepniewska

**Affiliations:** 1 WorldWide Antimalarial Resistance Network (WWARN), University of Oxford, Oxford, UK; 2 Centre for Tropical Medicine, Nuffield Department of Medicine, University of Oxford, Oxford, UK; 3 Mahidol-Oxford Tropical Medicine Research Unit, Faculty of Tropical Medicine, Mahidol University, Bangkok, Thailand; 4 Shoklo Malaria Research Unit, Mahidol-Oxford Tropical Medicine Research Unit, Faculty of Tropical Medicine, Mahidol University, Mae Sot, Thailand; 5 Laboratory of Malaria and Vector Research, National Institute of Allergy and Infectious Diseases, National Institutes of Health, Bethesda, Maryland, USA; 6 Center for Parasitology, Entomology and Malaria Control, Phnom Penh, Cambodia; 7 Kenya Medical Research Institute/Wellcome Trust Research Programme, Kilifi, Kenya; 8 Unit of Infectious Diseases, Department of Medicine Solna, Karolinska Institutet, Stockholm, Sweden; 9 Global Health, Department of Public Health Sciences, Karolinska Institutet, Stockholm, Sweden; 10 Lao-Oxford-Mahosot Hospital-Wellcome Trust Research Unit (LOMWRU), Microbiology Laboratory, Mahosot Hospital, Vientiane, Lao PDR; 11 Department of Immunology and Medicine, Armed Forces Research Institute of Medical Sciences (AFRIMS), Bangkok, Thailand; 12 Armed Forces Research Institute of Medical Sciences (AFRIMS), Phnom Penh, Cambodia; 13 Institute of Specific Prophylaxis and Tropical Medicine, Medical University of Vienna, Vienna, Austria; 14 Malaria Research and Training Centre, University of Bamako, Bamako, Mali; 15 Oxford University Clinical Research Unit (OUCRU), Ho Chi Minh City, Vietnam

**Keywords:** Plasmodium falciparum, Malaria, Artemisinin resistance, Parasite clearance, Simulation

## Abstract

**Background:**

The emergence of *Plasmodium falciparum* resistance to artemisinins in Southeast Asia threatens the control of malaria worldwide. The pharmacodynamic hallmark of artemisinin derivatives is rapid parasite clearance (a short parasite half-life), therefore, the *in vivo* phenotype of slow clearance defines the reduced susceptibility to the drug. Measurement of parasite counts every six hours during the first three days after treatment have been recommended to measure the parasite clearance half-life, but it remains unclear whether simpler sampling intervals and frequencies might also be sufficient to reliably estimate this parameter.

**Methods:**

A total of 2,746 parasite density-time profiles were selected from 13 clinical trials in Thailand, Cambodia, Mali, Vietnam, and Kenya. In these studies, parasite densities were measured every six hours until negative after treatment with an artemisinin derivative (alone or in combination with a partner drug). The WWARN Parasite Clearance Estimator (PCE) tool was used to estimate “reference” half-lives from these six-hourly measurements. The effect of four alternative sampling schedules on half-life estimation was investigated, and compared to the reference half-life (time zero, 6, 12, 24 (A1); zero, 6, 18, 24 (A2); zero, 12, 18, 24 (A3) or zero, 12, 24 (A4) hours and then every 12 hours). Statistical bootstrap methods were used to estimate the sampling distribution of half-lives for parasite populations with different geometric mean half-lives. A simulation study was performed to investigate a suite of 16 potential alternative schedules and half-life estimates generated by each of the schedules were compared to the “true” half-life. The candidate schedules in the simulation study included (among others) six-hourly sampling, schedule A1, schedule A4, and a convenience sampling schedule at six, seven, 24, 25, 48 and 49 hours.

**Results:**

The median (range) parasite half-life for all clinical studies combined was 3.1 (0.7-12.9) hours. Schedule A1 consistently performed the best, and schedule A4 the worst, both for the individual patient estimates and for the populations generated with the bootstrapping algorithm. In both cases, the differences between the reference and alternative schedules decreased as half-life increased. In the simulation study, 24-hourly sampling performed the worst, and six-hourly sampling the best. The simulation study confirmed that more dense parasite sampling schedules are required to accurately estimate half-life for profiles with short half-life (≤three hours) and/or low initial parasite density (≤10,000 per μL). Among schedules in the simulation study with six or fewer measurements in the first 48 hours, a schedule with measurements at times (time windows) of 0 (0–2), 6 (4–8), 12 (10–14), 24 (22–26), 36 (34–36) and 48 (46–50) hours, or at times 6, 7 (two samples in time window 5–8), 24, 25 (two samples during time 23–26), and 48, 49 (two samples during time 47–50) hours, until negative most accurately estimated the “true” half-life. For a given schedule, continuing sampling after two days had little effect on the estimation of half-life, provided that adequate sampling was performed in the first two days and the half-life was less than three hours. If the measured parasitaemia at two days exceeded 1,000 per μL, continued sampling for at least once a day was needed for accurate half-life estimates.

**Conclusions:**

This study has revealed important insights on sampling schedules for accurate and reliable estimation of *Plasmodium falciparum* half-life following treatment with an artemisinin derivative (alone or in combination with a partner drug). Accurate measurement of short half-lives (rapid clearance) requires more dense sampling schedules (with more than twice daily sampling). A more intensive sampling schedule is, therefore, recommended in locations where *P. falciparum* susceptibility to artemisinins is not known and the necessary resources are available. Counting parasite density at six hours is important, and less frequent sampling is satisfactory for estimating long parasite half-lives in areas where artemisinin resistance is present.

## Background

Anti-malarial drug resistance poses a serious threat to global efforts to control and eliminate malaria. During the 1980s and 1990s, malaria-related mortality increased due to the spread of *Plasmodium falciparum* resistance to anti-malarial drugs. This trend was reversed by replacing failing drugs with highly efficacious artemisinin-based combination therapy (ACT) and deployment of improved vector control measures. ACT is now the recommended first-line treatment for *P. falciparum* malaria in almost all endemic countries [1,2]. In the past, parasite resistance to chloroquine and then to sulphadoxine-pyrimethamine spread from Western Cambodia throughout Asia and Africa. The recent emergence of artemisinin resistance in *P. falciparum* malaria on the Thailand-Cambodia border therefore poses a considerable global health threat [3-5].

To date, the molecular basis of artemisinin resistance has not been elucidated and conventional *in vitro* drug response assays have provided conflicting results. Recently, a ring stage survival assay has been proposed, but it requires specific skills, training and validation [6,7]. Strong evidence of artemisinin resistance in Southeast Asia was recognized by the significant reduction in the parasite clearance rate following artesunate treatment and increased failure rates following ACT administration [4,8,9]. Until a definitive molecular marker is identified and validated in various regions, accurate and reliable measurement of parasite clearance remains a robust and simple method of assessing the spread or independent emergence of artemisinin-resistant *P. falciparum* in Southeast Asia and elsewhere. Furthermore, parasite clearance is becoming an essential component of the measurement of the efficacy of new anti-malarials.

Following treatment with an effective anti-malarial drug, the clearance of parasites from the peripheral blood is proportional to the parasite density (that is, a first-order process) [10,11]. As such, the predominant relationship between the log-transformed parasite density and time is generally linear [12-14]. The slope of the log-parasitaemia *versus* time relationship is considered the most robust measure of parasite clearance [15], and of various different *in vivo* measures shows the highest heritability among *P. falciparum* parasites in a setting where artemisinin resistance is prevalent [16]. However, several potential sources of error can be introduced if a straight line is fitted to all log-parasite density data [14]. Previous estimates of parasite clearance rates have, therefore, been complicated by observer subjectivity in how to handle these sources of variation [14,15].

To facilitate the standardized and accurate estimation of parasite clearance rates, the WorldWide Antimalarial Resistance Network (WWARN) previously developed the Parasite Clearance Estimator (PCE) tool, now available online [14,17]. The PCE expresses parasite clearance in terms of the slope half-life and has been used to quantify the clearance distributions in several studies (for example, [18-21]). Using this tool, parasite clearance distributions may be compared from different study locations and times, where half-life distributions that are centered around smaller half-lives correspond to a relatively sensitive response compared to distributions centered at longer half-life values. For example, the parasite clearance distribution in Pailin, Cambodia, in 2008–2010 showed evidence of slow clearance with a median half-life of 5.8 hours [21] while the distribution in Mali in 2010 showed evidence of a sensitive response (median half-life of 1.9 hours) [19].

In order to accurately estimate parasite clearance using the current version of the PCE tool, frequent parasite counts (at least twice daily) are recommended. However, most *in vivo* assessments published in the literature measure parasitaemia either daily, or only on days D0, D2 and D3, as recommended by the World Health Organization [22]. The proportion of patients with detectable parasitaemia on D3 is a simple measure of parasite clearance at the population level [23], and provides a useful metric of response that can be widely applied. However, “D3-positivity” at the individual patient level is inaccurate because it depends heavily on the pre-treatment parasite density and the precise timing of sampling, and these can vary substantially within and across clinical trials. D3 can correspond to a time ranging from 60 to 80 hours after treatment, depending on the time of patient enrolment and the subsequent follow-up visits. For example, a patient first treated at 09.00 on D0 may be assessed for parasitaemia at 17.00 on D3 (80 hours later).

To compare parasite clearance rates across different study sites and times, frequent parasite counts are needed to generate accurate and reliable estimates. This method is dependent on resource and operational constraints, capacity to generate quality-assured parasite density counts, and patient convenience (in terms of how often blood samples are taken), all factors that may limit the number of measurements that can be taken at a given site. In fact, the six hourly schedule was arbitrarily defined, and there have been no studies validating that these measurements need to be, or indeed should be, collected at regular time-intervals to best facilitate clearance estimation. Schedules that produce accurate rate estimates yet minimize patient (and/or their caretakers) inconvenience (including hospitalization and night-time blood collection) would clearly be preferable. Here, the effects of potential alternative sampling schedules on the estimation of parasite clearance rates were investigated using real patient data from 13 studies with a combined sample size of 4,652 patients, conducted between 2001 and 2011 in Cambodia, Thailand, Mali, Kenya and Vietnam.

## Methods

This paper used three approaches to investigate the effect of different sampling schedules on parasite clearance estimates. The first approach used a large dataset of patient data, pooled across 13 studies conducted between 2001 and 2011, to compare the HL estimates from four alternative sampling schedules. The second used statistical bootstrap method to investigate the effect on population estimates of HL when the same four alternative schedules were applied to populations that varied from short to very long geometric mean HL. In the final approach, a simulation study was designed in which parasite counts were generated so that more complicated sampling schedules could be assessed.

### Study data

This work was conducted by the Parasite Clearance Study Group, under the auspices of WWARN, as part of a larger effort to combine all available data from ACT efficacy studies that measured parasite densities at least twice daily. Parasite density-time profiles from published and unpublished studies that measured six-hourly parasite densities were sought for this analysis. Studies were identified among those that used the PCE tool and through calls for data at international meetings. Only studies in which patients were treated with artesunate alone or in combination with a partner drug were considered. Within each study, only patients with parasite densities measured every six hours until parasitaemia became undetectable (i.e., “negative”) on blood smears were included.

Thirteen datasets fulfilled these inclusion criteria and all had one of three sampling schedules (Table 1). The most common schedule was every six hours until negative (SS1). The second most common sampling method was zero, two, four, six, eight and 12 hours and then every six hours until negative (SS2). The schedule for one study (Thailand2) was zero, four, eight and 12 hours and then every six hours until negative (SS3). This latter study, which did not meet the inclusion criterion of reporting parasite density at six hours, was nevertheless included and the parasite density at four hours used in the analysis instead. Since the exact times of blood sampling varied slightly between studies, measurements made every six ± one hour were considered acceptable for inclusion. Seven studies counted parasites using both thin and thick smears, either per 1,000 red blood cells (RBC) and 500 white blood cells (WBC), 1,000 RBC and 400 WBC, 1,000 RBC and 200 WBC or 5,000 RBC and 200 WBC (Table 1). In five studies, parasites were counted per 200 WBC or 300 WBC in thick smears only. The actual times of blood sampling were recorded in all studies and were used in the present analysis to estimate parasite clearance.

**Table 1:** Summary of the 13 included studies

**Country**	**Years**	**Number patients**	**Sampling schedule**	**Counting method**		**DLM**	**Reference**
				**Thick (WBC)**	**Thin (RBC)**		
Thailand1	2001-2010	3391	SS1	500	1000	16	Phyo *et al.*[20]
Thailand2	2008	40	SS3	200	1000	16	Dondorp *et al.*[4]
Thailand3	2009-2010	80	SS2	200	1000	16	Das *et al.*[21]
Mali	2010	261	SS1	300	ND	25	Lopera-Mesa *et al.*[19]
Cambodia1	2007-2008	59	SS2	200	1000	16	Dondorp *et al.*[4]
Cambodia2	2008-2010	79	SS2	200	1000	16	Das *et al.*[21]
Cambodia3	2009	79	SS1	200	ND	15	Amaratunga *et al.*[18]
Cambodia4	2010	98	SS1	200	ND	15	Amaratunga *et al.*[18]
Cambodia5	2010	30	SS1	200	ND	15	Amaratunga *et al.*[18]
Cambodia6	2010	55	SS1	200	ND	15	Unpublished
Cambodia7	2008-2009	143	SS2	200	5000	14	Bethell *et al.*[24]
Kenya	2010-2011	171	SS2			30	Unpublished
Vietnam	2010-2011	166	SS1	400	1000	8	Hien *et al.*[25]
**Total**		**4652**					

SS1 = six-hourly until negative; SS2 = zero, two, four, six, eight and 12 hours, and then six-hourly until negative; SS3 = zero, four, eight and 12 hours, and then six-hourly until negative; ND = not done; DLM = detection limit of microscopy (in parasites per μL); RBC = red blood cells; WBC = white blood cells.

### Parasite clearance parameters

**Figure 1:**
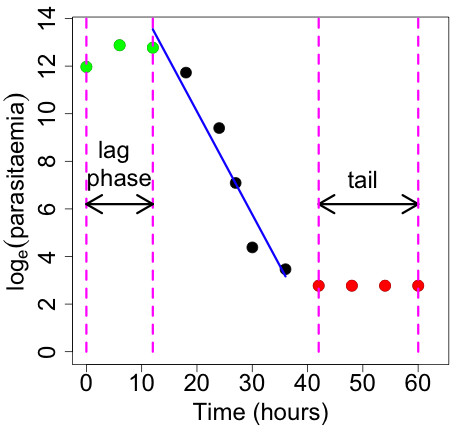
Schematic representation of the WWARN Parasite Clearance Estimator model of parasite clearance.

As a measure of parasite clearance, the parasite half-life (HL) was calculated for each patient’s parasite density-time profile, using WWARN’s PCE tool. A schematic of the parasite clearance profile is shown in Figure 1. The PCE selects the most appropriate model (linear, quadratic or cubic) to fit the log-transformed parasite densities. The model then detects when a “lag-phase” is present (ie, the initial part of the profile having a much flatter slope than the remaining part of the profile). The HL is then calculated, based on the slope of the linear part of the curve, adjusted for this lag-phase. The “tail” of the profile (if present), defined as the terminal part of the profile when parasitaemia remains close to the limit of detection and does not decrease over a number of time-points, is excluded before parasite clearance is estimated. Using this method, the parasite clearance estimate is independent of the initial parasite density since the rate of clearance is measured rather than the time to clearance. Departing from previous approaches, parasite counts below the limit of detection are accounted for using tobit regression [26] and are not excluded from the model. The PCE model is detailed in [14] and has been recently validated [16].

### Approach 1: Effect of alternative sampling schedules on estimation of half-life

A common sampling schedule used in clinical studies in the field is 12-hourly sampling; however, if the parasite clearance profile has a lag-phase, this schedule may be unable to detect it (Figure 1). The effect of adding additional observations in the first 24 hours to the 12-hourly sampling schedule was investigated. For each patient included in the analysis, a reference (“true”) HL was calculated using parasite densities measured every six hours until the first negative parasitaemia. Four alternative sampling schedules (Table 2) were investigated and the HL estimates obtained for these schedules were compared to the reference HL estimates. Alternative schedules A1, A2 and A3 included four parasite densities in the first 24 hours, excluding a measurement at 18, 12 or six hours, respectively. After 24 hours, data points were included every 12 hours until the first negative parasitaemia. Alternative schedule A4 used 12-hourly measurements for the whole sampling period. For each of the patient profiles, the relative difference in HL for each alternative sampling schedule was defined as:

$$\mathrm{HL}\;\mathrm{relative}\;\mathrm{difference}\;=\;\frac{\mathrm{SS}\_\mathrm{HL}\;‒\;R\_\mathrm{HL}}{R\_\mathrm{HL}}\;\times \;100\%,$$

where SS_HL and R_HL are the HL estimates for the alternative and reference sampling schedules, respectively. The difference in HL was defined as:

HLdifference=SS_HL‒R_HL.

**Table 2:** The sampling time-points (in hours) included in the reference schedule and each of the four alternative schedules, A1-A4, for the analysis of real patient data

**Sampling schedule**	**0**	**6**	**12**	**18**	**24**	**30**	**36**	**42**	**48**	**Every 6 hours until negative**	**Every 12 hours until negative**
Reference	X	X	X	X	X	X	X	X	X	X	
A1	X	X	X		X		X		X		X
A2	X	X		X	X		X		X		X
A3	X		X	X	X		X		X		X
A4	X		X		X		X		X		X

The proportions of profiles that were misclassified when the HL estimates were dichotomized using cut-off HL values of three, four, five and six hours were also reported. That is,

$$\begin{array}{cc} \mathrm{Proportion}\;\mathrm{misclassified} & =\mathrm{proportion}\;\mathrm{profiles}\;\mathrm{with}\;\mathrm{SS}\_\mathrm{HL}\\ & \;>\mathrm{cutoff}\;\mathrm{and}\;R\_\mathrm{HL}<\mathrm{cutoff}\\ & +\;\mathrm{proportion}\;\mathrm{profiles}\;\mathrm{with}\;\mathrm{SS}\_\mathrm{HL}\\ & \;<\mathrm{cutoff}\;\mathrm{and}\;R\_\mathrm{HL}>\mathrm{cutoff} \end{array}$$

### Approach 2: Sampling distribution of half-life with bootstrapping

To study the effect of different sampling schedules on population estimates of HL for different geometric mean HL, a bootstrapping algorithm with replacement (see for example, [27]) was adopted whereby 100 patients were randomly selected from the reference dataset to form a theoretical cohort and the sampling procedure was repeated 1,000 times. When 100 patients were selected from the reference dataset, the same 100 patients created the datasets for the four alternative schedules. The effect of each schedule was evaluated by examining the distribution of the median HL estimates and the proportion of profiles with a HL above three, four, five and six hours. Bootstrap samples were selected from the whole reference dataset itself so that the HL followed a pre-specified log-normal distribution (*HL* ~ logN (*μ,sd*^2^) as follows. Given the reference geometric mean HL (M) and the coefficient of variation (CV), the parameters of the log-normal distribution were calculated: $\mu =\log_{e}\left(M\right)\;\mathrm{and}\;\mathrm{sd}=\sqrt{\log_{e}\left(CV^{2}+1\right)}$. For each bootstrap sample, j = 1 to 1000, the following steps were completed:

a) segmented the reference HL distribution into discrete sections, S_i_, i = 1…r. The segments were each of one hour width from zero hours to 13 hours (the range of HL is 0.7-12.9).

b) selected one of the r segments from (a), chosen at random.

c) identified reference HLs from the real data that lie within the r^th^ segment, chosen in (b).

d) sampled one of the reference HLs identified in (c), chosen at random.

e) accepted the reference HL as the j^th^ bootstrap sample with a probability p, where p is the log-normal density for the given values of M and CV, for the HL chosen in (d).

f) repeated steps (a) – (e) until 100 patients had been accepted into the j^th^ bootstrap sample.

Geometric mean (M) HLs of two, three, four, five, six and seven hours and CVs of 20, 30, 40 and 50% were considered. For each combination of M and CV, 1,000 bootstrap samples (each of 100 patients) were sampled from the reference HL distribution (and subsequently from the alternative schedule HL distributions). The relative difference in median HL from the bootstrap samples for each alternative sampling schedule was defined as:

$$\begin{array}{l} \mathrm{relative}\;\mathrm{difference}\;\mathrm{in}\;\mathrm{median}\;\mathrm{HL}\;\\ \;=\;\frac{\mathrm{median}\;\mathrm{SS}\_\mathrm{HL}\;‒\;\mathrm{median}\;R\_\mathrm{HL}}{\mathrm{median}\;R\_\mathrm{HL}}\;\times \;100\%, \end{array}$$

where ‘median SS_HL’ and ‘median R_HL’ are the median HL estimates for the alternative and reference sampling schedules, respectively.

### Approach 3: Comparison of sampling schedules on simulated data

To investigate more complicated sampling schedules that included a time-point not represented in the reference schedule, a simulation study was designed. Parasite counts were generated based on the variability observed in the real patient data, so that the created profiles were realistic. The process by which parasite count data were generated, for a given HL and P0, is presented in Additional file 1. For a specified “true” HL and “true” initial parasitaemia (P0), 1,000 log-parasite density-time profiles were generated, and the HLs using a number of different sampling schedules (Table A2.1 in Additional file 2) were calculated using the PCE tool. These include regular sampling schedules (S1-S4), the best sampling schedule from the bootstrap analysis (B1 = A1), an optimal sampling schedule [28], schedules based on once daily repeated sampling (M1-M3) and slight modifications of these schedules. All combinations of “true” HLs of two, three, four, five and six hours and “true” initial parasite densities (P0) of 5,000, 10,000, 50,000, 100,000, and 200,000 per μL were examined. The effect of the sampling schedules on estimates of HL was assessed through comparisons with the “true” HL using the proportion of profiles with an absolute value of relative difference (ARD) more than 10, 20 and 30% and the proportion of profiles with an absolute value of difference (AD) greater than one hour. For the two sampling schedules that stopped at 48 hours (O1 and S1c), large values of the HL and high P0 combinations could result in a final parasite density exceeding 1,000 per μL, in which case a HL estimate is not typically available through the PCE tool [14]. To facilitate comparison of these two schedules, the PCE tool was adjusted and run separately for these two schedules such that the HL was estimated, regardless of what was the parasite density at 48 hours.

## Results

### Study data

The 13 studies that satisfied the inclusion criteria (Table 1) have a combined sample size of 4,652 patients, and were conducted in Cambodia (n = 7), Thailand (n = 3), Mali (n = 1), Kenya (n = 1) and Vietnam (n = 1) between 2001 and 2011. The sample size and detection limit of microscopy for each study are summarized in Table 1. In the 13 studies, 2,746 patients satisfied the inclusion criteria of regular six-hourly parasite density counts and were included in the reference dataset and analysis (Table 3).

**Table 3:** Summary of results from 13 included studies

**Country**	**Proportion of patients**	**Median HL; range; IQR**	**CV (%)**	**Std dev log(HL)**	**Prop NZ tlag**	**Median NZ tlag**	**Prop with tail**
Thailand1	46% (1571/3391)	3.05; 0.889-12.9; 2.37-4.24	46.4	0.438	26.4% (n = 415)	6	42.1% (n = 662)
Thailand2	82% (33/40)	2.87; 1.48-7.54; 2.11-3.77	46.8	0.421	18.2% (n = 6)	4.05	54.5% (n = 18)
Thailand3	88% (70/80)	3.26; 0.964-9.09; 2.36-4.42	50.4	0.49	24.3% (n = 17)	6	41.4% (n = 29)
Mali	99% (258/261)	1.87; 0.678-4.27; 1.57-2.34	30.0	0.296	58.1% (n = 150)	6	1.55% (n = 4)
Cambodia1	97% (57/59)	6.11; 2.53-9.5; 4.93-7.18	26.1	0.285	21.1% (n = 12)	12	45.6% (n = 26)
Cambodia2	94% (74/79)	5.79; 2.07-9.37; 4.85-7.32	31.1	0.359	24.3% (n = 18)	6.02	40.5% (n = 30)
Cambodia3	99% (78/79)	6.09; 1.71-11.2; 4.69-7.15	32.4	0.377	7.69% (n = 6)	6	14.1% (n = 11)
Cambodia4	100% (98/98)	6.5; 2.13-11.3; 5.14-7.73	29.2	0.329	6.12% (n = 6)	15	2.04% (n = 2)
Cambodia5	100% (30/30)	5.75; 2.66-10.5; 4.12-7.48	34.5	0.363	33.3% (n = 10)	15	13.3% (n = 4)
Cambodia6	78% (43/55)	2.62; 1.13-4.43; 2.31-3	25.9	0.278	6.98% (n = 3)	6	27.9% (n = 12)
Cambodia7	98% (140/143)	7.03; 1.7-11.8; 5.6-8.11	31.6	0.397	6.43% (n = 9)	6	57.1% (n = 80)
Kenya	93% (159/171)	2.49; 0.956-5.19; 1.89-3.09	33.1	0.354	19.5% (n = 31)	6	0% (n = 0)
Vietnam	81% (135/166)	2.9; 1.02-10.2; 2.04-5.46	59.3	0.572	34.1% (n = 46)	6.23	40.7% (n = 55)
**Total**	**59% (2746/4652)**	**3.13; 0.678-12.9; 2.29-5**	**53.8**	**0.519**	**26.5% (n = 729)**	**6 (6–6.25)**	**34% (n = 933)**

For each of the studies, country; proportion of total patients included; median HL (range; IQR); coefficient of variation (CV) as a percentage; standard deviation of log (HL); proportion of profiles with a non-zero tlag; the median tlag for the profiles with a non-zero tlag; and the proportion of profiles with a tail. The bottom row gives the corresponding values for the pooled dataset. IQR = interquartile range; NZ = non-zero; prop = proportion.

### Parasite clearance parameters

The median, range and interquartile range (IQR) of parasite HLs for individual studies and all studies combined are presented in Table 3. The median (range) HL of all studies was 3.1 hours (0.7-12.9) and varies between studies, ranging from 1.9 hours to 7.0 hours. The proportion of profiles with a non-zero lag-phase (tlag) was 26.5% for all studies, and ranged from six to 58% (Table 3). Of those profiles with a non-zero tlag, the median lag-phase duration of all studies was six hours, and ranged from four to 15 hours. The coefficient of variation of HL estimates for all studies was 54%, and ranged between 26 and 59%. The proportion of profiles showing a tail (i.e., the terminal part of the profile when parasitaemia remains close to the detection limit) for all studies was 34% and between 0 and 57% in individual studies.

### Approach 1: Effect of alternative sampling schedules on estimation of half-life

The effect of using alternative schedules A1, A2, A3 and A4 to estimate parasite clearance was assessed first on the 2,746 real patient profiles. The median (IQR) difference in HL estimates between the reference and alternative sampling schedules was 0.01 (-0.15-0.21), 0.06 (-0.10-0.29), 0.09 (-0.09-0.34) and 0.15 (-0.06-0.46) hours, for A1, A2, A3 and A4, respectively (Table 4). Overestimation of the HL by the alternative schedule (i.e., when the HL from the alternative schedule exceeded the reference HL) occurred in 50, 60, 60 and 66% of profiles using the A1, A2, A3 and A4 schedules, respectively. If there were no systematic bias in the estimation of HL, then on average overestimation would occur in 50% of samples. A zero lag phase (tlag) under the alternative schedule and a difference in the use of tobit regression were both causes of overestimation of the HL under the A1-A4 schedules, compared to the reference schedule (Table A3.1, Additional file 3).

**Table 4:** Summary of results from reference and alternative schedules

**Schedule**	**Prop OE (of 2746 profiles)**	**(IQR)**	**CV (%)**	**Std dev log(HL)**	**Prop NZ tlag**	**Median (IQR) NZ tlag**	**Prop tail**	**Prop (%) profiles misclassified, with HL cutoff of:**			
								**3 h**	**4 h**	**5 h**	**6 h**
Ref	0%	0 (0–0)	54.5	0.52	26.5 (n = 729)	6 (6–6.25)	34% (n = 933)	0	0	0	0
A1	49.9%	0.01 (-0.15-0.21)	51.6	0.503	22.1 (n = 606)	6 (6–6.27)	9.36% (n = 257)	7.7	3.9	2.7	2.2
A2	60.2%	0.06 (-0.10-0.29)	51.1	0.482	17.4 (n = 478)	6 (6–6.03)	9.87% (n = 271)	8.6	4.4	3	2.2
A3	59.8%	0.09 (-0.09-0.34)	50.9	0.484	12.5 (n = 344)	12 (12–12)	9.91% (n = 272)	9.5	4.5	3	2.1
A4	66.3%	0.15 (-0.06-0.46)	47.7	0.435	6.96 (n = 191)	12 (12–12)	9.25% (n = 254)	10	4.6	3.4	2.2

For each of the reference and alternative schedules (A1-A4) applied to real patient data: the proportion of profiles that overestimate the HL (relative to the reference HL); median difference in HL (IQR); coefficient of variation of HL (CV) as a percentage; standard deviation of log (HL); the proportion of profiles with a non-zero lag phase (tlag); the median (IQR) tlag for profiles with a non-zero tlag; the proportion of profiles with a tail and the proportions of profiles that are misclassified when the HL estimates are dichotomized using HL cut-offs of three, four, five and six hours. IQR = interquartile range; Ref = reference; OE = overestimation (proportion of HLs from alternative schedule which exceeded reference HL); NZ = non-zero; prop = proportion.

The 12-hourly schedule (A4) was the worst performing schedule when the real patient data were used. The main determinants of the discrepancy between the reference and A4 schedules were the presence of a lag-phase in the reference profile and a small number of measurements, which resulted in a simple regression model fitted for the A4 schedule (with the first zero replaced by the detection limit) as opposed to a tobit regression fitted for the reference data (Table A3.2, Additional file 3). A six-hour delay in the time when the first negative parasitaemia was recorded (when using A4 *versus* the reference schedule) did not matter overall.

**Figure 2:**
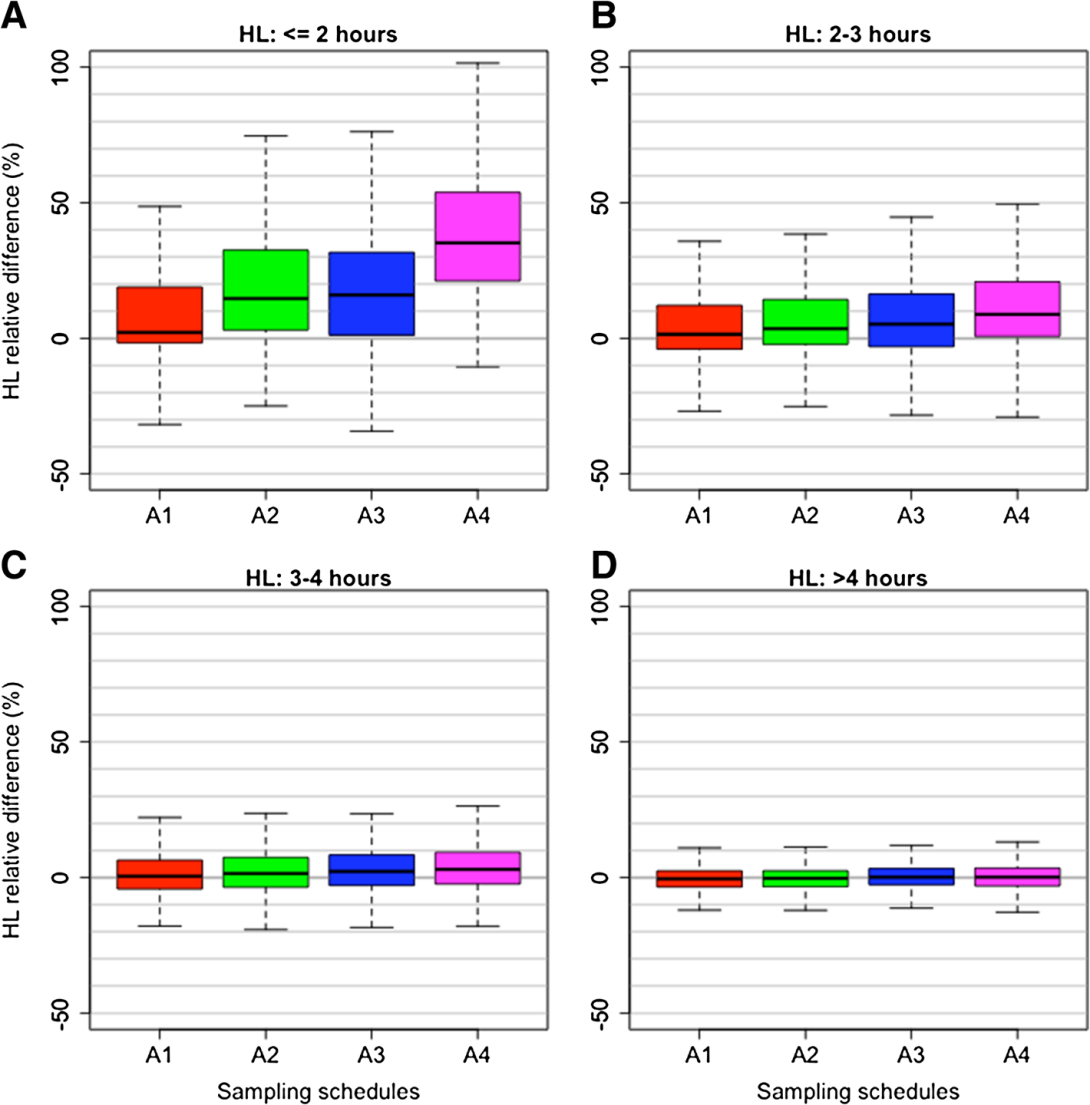
**Relative difference (%) in half-life between alternative schedules A1-A4 and the reference dataset (six-hourly sampling), using actual study data.** See Table 1 for a list of 13 included studies. Boxplots show the median, interquartile range and range. Panels **A**, **B**, **C** and **D** correspond to HLs less than 2 hours, between 2-3 hours, between 3-4 hours and more than 4 hours, respectively.

The difference between HLs estimated by the alternative and reference schedules was greater for profiles with a short reference HL (compare HL > 4 and HL ≤ 2 in Figure 2) and could have a considerable effect on the estimates of the distribution of HL in a study. The misclassification of profiles was highest for a cutoff value of HL of three hours (7.7, 8.6, 9.5 and 10%, respectively for A1, A2 A3 and A4) (Table 4).

### Approach 2: Sampling distribution of half-life with bootstrapping

**Figure 3:**
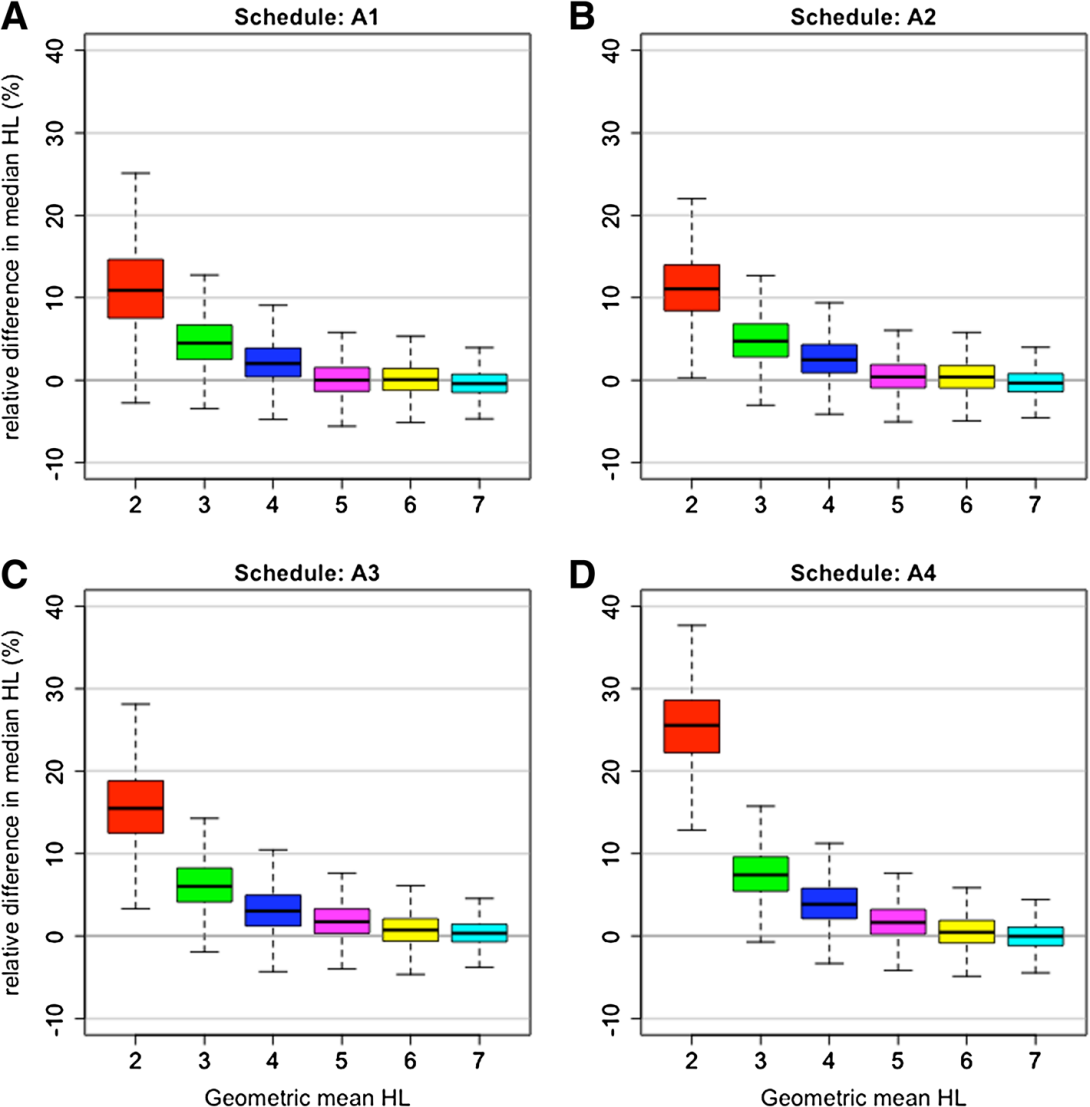
**Relative difference (%) between median half-life from alternative schedules A1-A4 and the reference dataset (six-hourly sampling) for the bootstrap samples, stratified by geometric mean half-life (two, three, four, five, six and seven hours).** Boxplots show the median, interquartile range and range. Panels **A**, **B**, **C** and **D** correspond to alternative schedules A1, A2, A3 and A4, respectively.

The effect of using alternative schedules A1-A4 on population estimates of HL from a log-normal distribution with pre-specified geometric mean HL and coefficient of variation was assessed using bootstrap methods. Overall, the alternative sampling schedules overestimated (median (range) relative difference) the median HL by 2.3% (-9.1-40.6). Here overestimation refers to the proportion of bootstrap samples in which the median HL from an alternative schedule exceeded the median HL from the reference schedule. Overestimation increased significantly in the 12-hourly schedule (A4) and in bootstrap datasets with a geometric mean HL of two hours (Figure 3). In this worst-case scenario (i.e., A4 schedule, HL = 2 hours), the median (range) relative difference in median HL was 26.0% (9.1-40.6) (Figure 3D). Estimation of the median HL was not affected by the CV of the datasets.

Examining the estimates of the proportion of profiles with a HL above a value of three, four, five and six hours, of all the alternative schedules applied to all bootstrap datasets, 7,981 bootstrap datasets gave different estimates of these proportions by absolute 10% difference or more. Of these, 7,224 (91%) occurred when the HL was above three hours. Among these profiles, 69% were for bootstrap samples with a geometric mean HL of two hours and 30% for a geometric mean HL of three hours, and <1% for all other HL.

**Figure 4:**
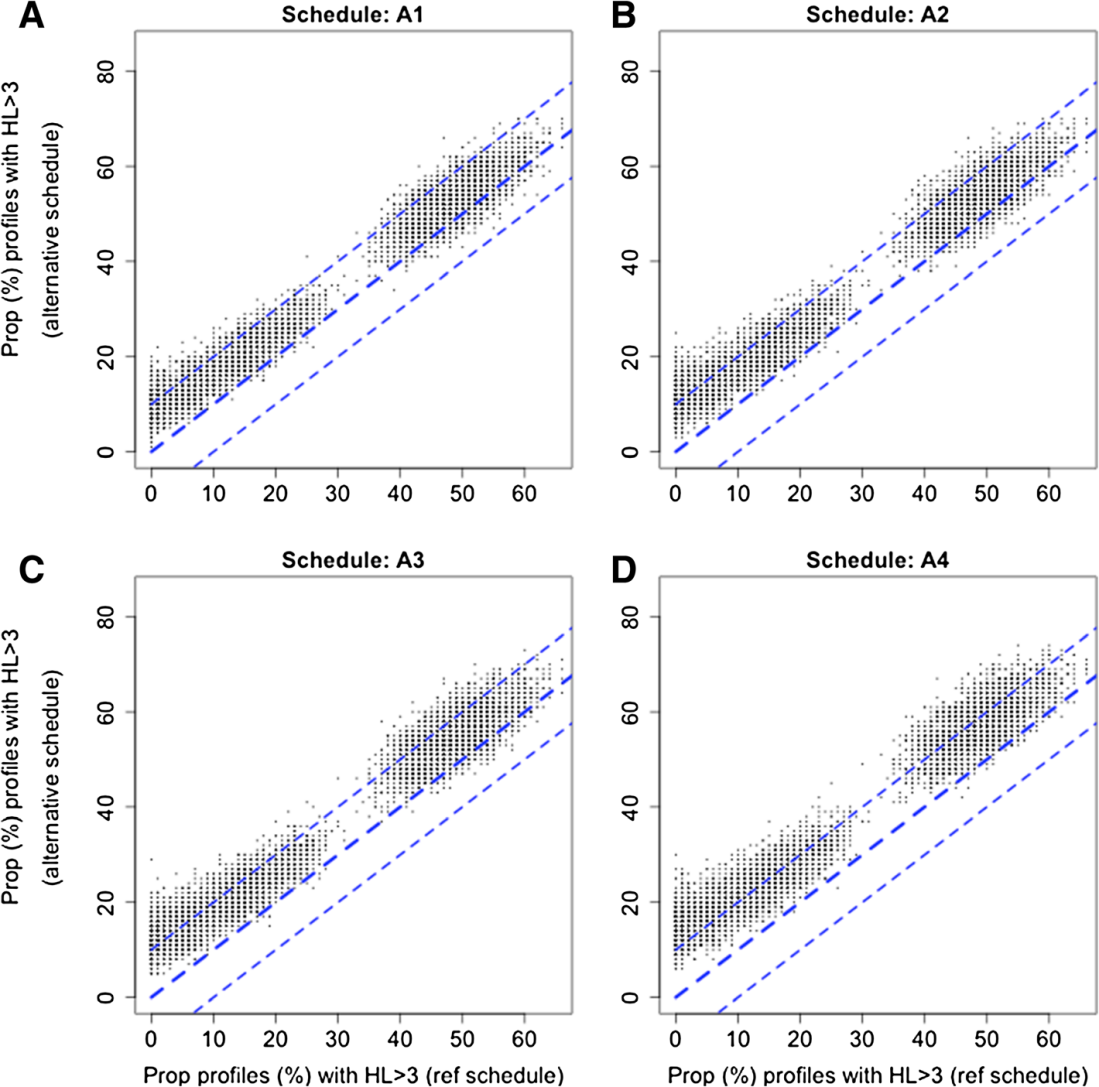
**The proportions of profiles with half-life (HL) more than three hours (in the bootstrap samples with a geometric mean HL = 2 or HL = 3 hours) from the alternative schedules A1-A4 compared with the proportion of profiles with HL > 3 hours from the reference six-hourly schedule.** The blue dashed lines represent y = x, y = x + 10 and y = x-10 lines. Panels **A**, **B**, **C** and **D** correspond to alternative schedules A1, A2, A3 and A4, respectively.

The 12-hourly schedule (A4) was also the worst performing schedule in the bootstrap analysis. The 12-hourly A4 schedule overestimated the proportion of profiles with a HL above three hours by 10% or more in 56% of bootstrap samples with a geometric mean HL of two hours and 31% for a geometric mean HL of three hours. These proportions were significantly lower for the other three alternative schedules: 13 and 4% for A1; 22 and 6% for A2 and 33 and 13% for A3. Estimation was only slightly affected by the CV. Comparing the proportions of profiles with a HL greater than three hours (for geometric mean HL of two or three hours) in the four alternative schedules (compared to the reference schedule) from each of the bootstrap samples shows the largest discrepancy of more than 10% overall for the 12-hourly A4 schedule (Figure 4).

### Approach 3: Comparison of sampling schedules on simulated data

In the final approach, a simulation study was designed to assess the effect of more complicated sampling schedules on parasite clearance estimation. Table A3.3, Additional file 3 summarizes the performance of the 16 sampling schedules in the simulation study, with results pooled for all values of HL and P0 considered. That is, for each schedule 25,000 profiles were used to create the summary statistics in Table A3.1, Additional file 3 (1,000 from each combination of HL and P0). For each simulation schedule, the proportion of profiles with AD (absolute value of the difference) more than one hour between the HL estimate under the schedule and the “true” HL, and the proportion of profiles with ARD (absolute value of relative difference) more than 10, 20 and 30% were calculated.

The worst-performing schedules, in terms of the highest ARD, were S4, M1 and S3, having 66, 53 and 52% of profiles with ARD > 10%; 49, 31 and 26% of profiles with ARD > 20% and 38, 19 and 13% with ARD > 30% (Table A3.3, Additional file 3). The best-performing schedule, in terms of the lowest ARD, for all HL and P0 values was the six-hourly schedule (S1). The eight-hourly sampling schedule (S2) had a consistently higher number of discrepant profiles (ARD > 10%) than S1, but the difference in the proportion of profiles with discrepant estimates was never more than 6%. More dense sampling than every 12 hours (S3) was required in the first 24 hours to accurately estimate HL for profiles with short HL and/or low initial parasite density. For HL ≤ 3, ARD > 20% was 19 and 34% for S1 and S3, respectively, compared to 14 and 20% for HL ≥ 4. Similarly for P0 ≤ 10,000 ARD > 20% was 24 and 39% for S1 and S3, respectively, compared to 11 and 17% for P0 ≥ 100,000. Among modified schedules (M1, M2 and M3), M2 had lower ARD > 20% and ARD > 30% than M1 while M2 performed better or similarly as M3, in terms of ARD > 20% and ARD >30%, except in simulations where HL = 2 and P0 ≤ 10,000.

For all schedules, except those involving 12 or 24-hourly sampling (i.e., S3, S4, M1, M2 and M3), the proportion of profiles with an absolute value of difference (AD) greater than one hour between the estimated HL and “true” HL increased with HL, but decreased with the initial parasitaemia. On the other hand, the proportion of profiles with an absolute value of the relative difference (ARD) greater than 10% (or 20%), was generally highest for a “true” HL of two hours and then remained constant or slightly decreased with HL. For schedules except S4, M2, and M3 the ARD slightly decreased with initial parasitaemia, but this effect was much less pronounced than for AD.

Among schedules in the simulation study with six or fewer measurements in the first 48 hours, when HL ≤ 4, the best performing schedules included B1, O1 and M2. Overall, the B1 and M2 schedules performed slightly better than O1, with 20, 19 and 22% of profiles with ARD > 20%, respectively. The three schedules gave similar results for slow clearing profiles, but not in the case of very fast clearance. For HL = 2, M2 had the lowest proportion of profiles with ARD > 10% (37 *versus* 52 for B1 and 52% for O1), and ARD > 20% (19 *versus* 25 and 26%) but similar proportions of profiles with ARD > 30% (13 *versus* 12 and 12%). The three schedules gave comparable results for high initial parasitaemia, but for low initial parasitaemia M2 performed better: ARD > 20% for M2, B1 and O1 were 22, 31 and 33% (P0 = 5,000), respectively compared to 19, 18 and 19% (P0 ≥ 10,000).

Continued sampling after 48 hours was important for slow clearing profiles. All six-hourly schedules (S1, S1a, S1b, S1c) gave the same results for HL ≤ 3, due to very few positive parasitaemias values recorded after 48 hours. The six-hourly schedules with 12-hourly (S1a) and six-hourly (S1) measurements after 48 hours gave similar results for longer HLs, while S1b and S1c performed worse. For HL ≥ 4, for example, the proportion of discrepant profiles for S1a and S1 were, 43 and 41% (ARD > 10%) and 16 and 14% (ARD > 20%), respectively. For S1b and S1c, these values increased to 46 and 45% (ARD > 10%); and 18 and 18% (ARD > 20%), respectively. When the optimal schedule (O1) was extended beyond 48 hours, the O1a (12-hourly) schedule gave slightly better estimation results while the O1b (24-hourly) schedule gave comparable results. All three schedules gave nearly identical results for HL ≤ 3 hours (ARD > 10% and ARD > 20% always within 1%) while for HL ≥ 4 hours there was a noticeable difference: the ARD > 20% was 22, 19 and 22% for the O1, O1a and O1b schedules, respectively, and 48, 45 and 49% for ARD >10%.

When the restriction of the final parasite density being less than 1,000 per μL was removed from the PCE algorithm, it was clear that the accuracy of the O1 and S1c schedules was poor if the measured parasitaemia at 48 hours was more than 1,000: ARD > 20% was 68% for O1 when the parasite density at 48 hours exceeded 1,000 compared to 33% for O1a. When the parasite density at 48 hours was less than 1000 the ARD > 20% was comparable: 24 and 19% for O1 and O1a, respectively. The same trend was observed for S1c versus S1: when the parasite density at 48 hours exceeded 1,000 ARD > 20% was 64 and 24% for S1c and S1, respectively (compared to 20 and 16% when the parasite density at 48 hours was less than 1,000).

## Discussion

**Figure 5:**
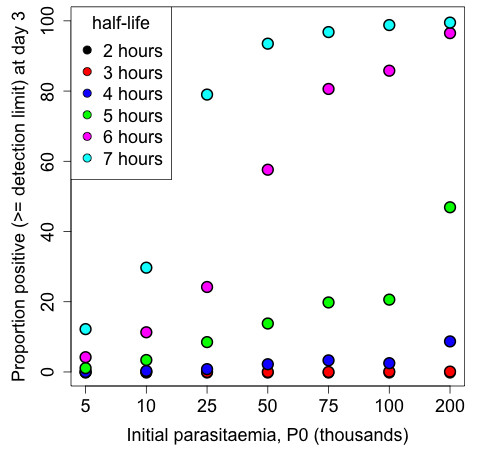
**The proportion of profiles at day 3 with a positive parasite count (more than or equal the limit of detection) as the initial parasitaemia (P0) varies from 5,000 to 200,000 based on 1,000 patients generated for each HL and P0 combination, as per the simulation study.** The black, red, blue, green, magenta and cyan points represent HLs of 2, 3, 4, 5, 6 and 7 hours, respectively.

**Figure 6:**
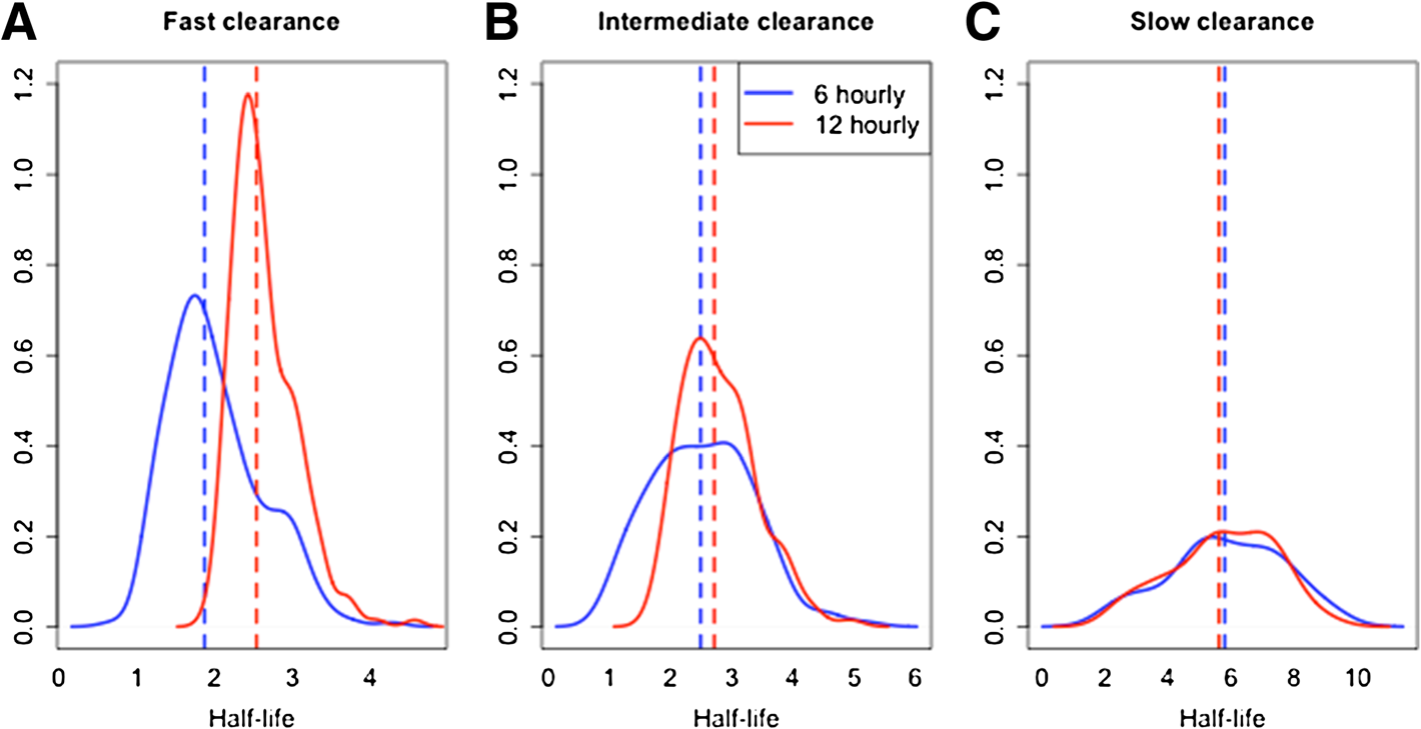
**Half-life distribution estimates for three datasets with fast, intermediate and slow clearance (subplots A, B and C, respectively), for the reference (six-hourly, blue) and the alternative A4 (12-hourly, red) sampling schedule.** The median HL for each study is shown with the vertical dashed line (A4 schedule in red, reference schedule in blue). Panels **A**, **B** and **C** correspond to studies with fast, intermediate and slow clearing parasites, respectively.

Surveillance of patient responses to ACT treatment is an important facet of the WHO Global Plan for Artemisinin Resistance Containment [4]. The proportion of patients who remain parasitaemic three days after treatment is a convenient metric that can warn of possible artemisinin resistance. However, in depth examination of the rate of parasite clearance in patients is needed in the context of efficacy trials of artemisinin derivatives, to determine whether the slow clearing phenotype is present and to detect early changes in parasite clearance. Figure 5 shows that for HL ≥ 5 hours, the proportion of D3 positive patients provides a clear indication of diminished response to artemisinin even with patients with modest initial parasite densities. However, if the HL is 4 hours, a measure that is useful as a very early sign of poor response, the proportion of patients still parasitaemic at day 3 is elevated only in patients with very high initial parasite loads. In order to provide very early detection of diminished artemisinin susceptibility, frequent parasite counts are required to accurately and reliably estimate parasite clearance rates, and this is especially true if the goal is to compare the rates of parasite clearance across different study sites and times. In this study, the effect of different sampling schedules on the estimation of parasite HL was investigated using three approaches: analysis of real patient data, a bootstrapping algorithm and a simulation study. Using frequent sampling data from 13 clinical studies (Table 1), HL estimates from four alternative schedules A1-A4 were calculated and then compared to the reference HL estimated from six-hourly data (Table 2). Compared to the reference schedule, the A1 schedule (zero, six, 12 and 24 hours, then 12-hourly) performed the best of the four alternative schedules, while the A4 schedule (12-hourly) performed the worst; however, this difference in performance was most obvious for populations with generally fast clearing parasites, and lower HLs (Figure 2). Thus, it was concluded that more frequent sampling is required to define these fast-clearing populations (Figure 6).

A bootstrapping algorithm was used to study the effect of different sampling schedules on population estimates of HL, for populations with different geometric mean HLs and coefficients of variation. Schedule A1 consistently performed better than the other schedules over a range of different geometric mean HLs. Its superiority was greater for profiles with shorter HLs (Figure 3). The 12-hourly A4 schedule performed the worst (Figure 3).

To investigate more complicated sampling schedules that included a time-point not present in the reference schedule, a simulation study was performed using “true” HLs of two, three, four, five and six hours and initial parasite densities (P0s) of 5,000, 10,000, 50,000, 100,000 and 200,000 per μL. The schedules which performed worst were 24-hourly sampling (S4), 12-hourly sampling (S3) and the modified 24-hour schedule (M1) (Table A3.1, Additional file 3), and the best-performing schedule was six-hourly sampling (S1). The simulation study confirmed that more dense parasite sampling schedules are required to accurately estimate half-life for profiles with short half-life (≤3 hours) and/or low initial parasite density (≤10,000 per μL). Among schedules in the simulation study with a limited number of measurements, when HL ≤ 4 hours, the best performing schedules included B1, O1 and M2.

The O1 schedule was identified by Jamsen *et al.*[28] as an optimal sampling schedule through a different methodological approach, and this was also investigated in the present simulation study. Jamsen *et al*. used a robust T-optimal design methodology to allow for discrimination across models that best describe an individual patient’s parasite-time profile. The design was based on the constraint that no more than six samples would be taken per patient within 48 hours of initial treatment. The T-optimal sampling times (windows) were: 0 (0–1.1), 5.8 (4.0-6.0), 9.9 (8.4-11.5), 24.8 (24.0-24.9), 36.3 (34.8-37.2) and 48 (47.3-48.0) hours after treatment initiation. It is interesting and lends support to our results that a sampling schedule driven by practicalities (B1) turned out to be a variant of a schedule identified by optimal design theory (O1).

In the simulation study, sampling after two days was found to give little additional improvement in parasite clearance HL estimates for fast clearing parasites (HL ≤ 3 hours). This observation is supported by the work of Nkhoma *et al.*[16], a study in which the majority of the profiles had cleared or reached very low parasitaemia levels by 48 hours. The reduced sampling schedule M2 performed very well in the simulation study; however, further investigation of this schedule in the field is required before it can be recommended widely. The simulation results will be extended to examine factors affecting HL estimates (eg, the thick and thin smear counting method combinations) and patient factors that affect clearance, notably the potential interaction between parasite clearance and clinical immunity. It is also important to recognize that high quality quantitative microscopy to measure the parasite density in the peripheral blood is essential for accurate estimation of parasite clearance rates.

The clinical phenotype of slow *P. falciparum* clearance is likely to remain a critical indictor of artemisinin resistance to be correlated with molecular markers when they are identified and validated. Furthermore, in the context of detecting the emergence and spread of artemisinin resistance, it is important to establish reliable baselines of parasite clearance for the different ACT available. Indeed, depending on the type and dosage of artemisinin derivatives in the various ACT (eg, artemether 1.7 mg/kg body weight in fixed-dose combination (FDC) of artemether-lumefantrine per dose; artesunate 4 mg/kg in FDC of artesunate-amodiaquine per day; and dihydroartemisinin 2 mg/kg in FDC of dihydroartemisinin-piperaquine), and the impact of the partner drugs, parasite clearance profiles may vary substantially. Hence, accurate and reliable estimation of parasite clearance rates is essential to monitor changes in artemisinin susceptibility within and between malaria-endemic regions. In investigating the effect of different sampling schedules on the accuracy of HL estimation, this study has shown that HL is best estimated by including samples at six and 12 hours (A1), and that 12-hourly sampling may be satisfactory in patients with slow-clearing parasites. It is particularly challenging to accurately estimate the HL in profiles with fast parasite clearance and low initial parasite density, the conditions usually encountered in high-transmission areas where individuals have significant levels of immunity.

## Conclusions

This study reveals important insights on sampling designs for accurate and reliable estimation of *P. falciparum* HL following treatment with artesunate alone or in combination with a partner drug. Including a parasite measurement at six hours is important, especially in regions with unknown *P. falciparum* susceptibility to artemisinins. Schedules with measurements at times (windows) of 0 (0–2), 6 (4–8), 12 (10–14), 24 (22–26), 36 (34–36) and 48 (46–50) hours, or at six, seven (two samples from 5–8), 24, 25 (two samples from 23–26), 48 and 49 (two samples from 47–50) hours, until negative are recommended. If the measured parasitaemia at two days exceeds 1,000 per μL, continued sampling at least once a day is suggested. A measure at 72 hours should be considered if the goal is to assess drug efficacy overall, and to conform to existing recommendations.

## Competing interests

The authors declare they have no competing interests.

## Authors’ contributions

KS, PJG and NJW designed the study. JAF implemented the algorithms and drafted the initial manuscript. JAF and KS analysed the data. KS, NJW, PJG and RMF edited the manuscript. FN, EAA, AMD, RMF, DS, SB, AB, AM, MM, PNN, DB, YS, MD, HN, AAD and TTH provided data and reviewed the manuscript. All authors read and approved the final version of this manuscript.

## Supplementary Material

Additional file 1 Additional methods. Click here for file

Additional file 2 Time-points for sampling schedules investigated in simulation study. Click here for file

Additional file 3 Additional results. Click here for file
