# Supplementary material for: Optimal sampling designs for estimation of Plasmodium falciparum clearance rates in patients treated with artemisinin derivatives
Source: Malar J. 2013 Nov 13;12:411. doi: 10.1186/1475-2875-12-411 (PMC3842737; doi:10.1186/1475-2875-12-411)
Supplement: Additional file 1 — Additional methods. [file 1475-2875-12-411-S1.pdf]

Additional file A1. Additional methods

This document describes the simulation study for generating patient profiles with a given initial parasitaemia (P_0) and half-life (HL).

A1.1. Procedure to generate parasite profile data

In line with the real data available, 26% of profiles were generated with a non-zero lag phase and the remaining profiles with a zero lag phase. In the case of a zero lag phase, the parasite counts were generated from a linear model:

$$Ptrue(t) = \exp(\log(P_0) - \log(2)/HL*t)$$

where P_0 is the intercept parasitaemia, HL is the half-life and t is time.
In the case of a nonzero lag phase, the parasitaemia were generated from a cubic model

$$Ptrue(t) = \exp(\log(P_0) + k_1t + k_2t^2 + k_3t^3)$$

Where P_0 is the intercept parasitaemia and k_1, k_2, k_3 are the coefficients of t, t^2 and t^3, respectively.

Step 1. Set parameters for the simulation

- standard deviation: s = 0.72 (chosen empirically from residuals of reference dataset)
- counting parameters: hct = 37, rper = 1000, wper = 500, minpara = 16
- number of patients: N = 1000
- proportion of tlag >0 patients: prop_tlag = 0.26 (chosen empirically from reference dataset)
- the final time at which parasite counts are to be collected: tend = 100
- the times at which parasite counts are to be collected: times = 1,2, 3, …, tend hours
- given a half-life = HL and initial parasitaemia = P_0

Step 2. Define the sampling schemes of interest:

- Reference schedule: every 6 hours
- Alternative schedules: see Table A2.1 for details

Step 3. Create model of cubic profile parameters, from reference dataset

- extract the cubic parameters from real patient data: intercept, k_1, k_2, k_3, final_slope (exclude those profiles for which a cubic model could not be fitted and those for which the lag phase exceeds 12 hours). The final_slope is the slope in the linear model that is the final model fitted to the data (in the PCE model), once the tail and lag phase have been identified.
- sample mean and covariance matrix are found for parameters (intercept, k_1, k_2, k_3 and final_slope) under the assumption of multivariate normal relationship between the parameters
- find the mean vector and covariance matrix for the conditional distribution of k_1, k_2, k_3, given a final_slope (-log(2)/HL) and intercept ($\log(P_0)$).

Step 4. Sample cubic parameters

- for prop_tlag*N patients, sample k_1, k_2 and k_3 from the conditional distribution in Step 3. Use rejection sampling to discard and replace any profiles with: i) a maximum parasitaemia > $3x10^6$, ii) a profile types that does not permit tlag >0, iii) a minimum parasitaemia > 1000, iv) monotonically increasing parasitaemia over input time.

Step 5. Generate patient data

Step 5.1. Generate patient data for patients without a lag phase (i = 1,…, (1-prop_tlag)*N)

- set the true parasitaemia, ptrue, to be the exponential of linear decline from the initial value of $\log(P_0)$, with a linear slope of -log(2)/HL (e.g. the same as final_slope in Step 3) over the time domain (times):

$$Ptrue(t) = \exp(\log(P_0) - \log(2)/HL*t)$$

Step 5.2. Generate data for patients with a lag phase (i = (1-prop_tlag)*N+1, …, prop_tlag*N)

- set the true parasitaemia, ptrue, to be the exponential of cubic profile from the ith sampled cubic parameters (k_3, k_2, k_1, $\log(P_0)$) from Step 4, over the time domain:

$$Ptrue(t) = \log(P_0) + k_1 t + k_2 t^2 + k_3 t^3$$

- find the time of the minimum parasitaemia, tmin:
  - * if turning points do not exist or the local minimum is outside of the time domain (times) or the cubic parasitaemia tends to negative infinity as time tends to infinity ($k_3<0$), then take the time of the minimum parasitaemia, tmin, as the final time, tend
  - * if there are multiple parasite values below the detection limit, but then it rises (because $k_3 >0$ and a local minimum exists in the time domain), then take the last value of time where the parasitaemia is below the detection limit as tmin.
  - * otherwise, set tmin to be the location of the local minimum.
  - -biologically implausible increases of ptrue after tmin are replaced with a tail. If two successive 0's are recorded, then the remaining parasite counts after this are set to 0.

Step 5.3. Calculate red and white blood cell counts

- calculate redcounts and whitecounts, based on the deterministic relationships:

redcount = Ptrue*rper/(hct*125.6*1000)

whitecount = Ptrue*wper/8000

The above equations are derived from the standard formulae, used in clinical studies to estimate the number of parasites/microlitre from the observed count of parasite per slide, namely:

Parasties/microliter = count per x rbc *125.6 *hct *1000/x (where x is the number of red blood cells)

Parasites/microliter = count per x wbc *8000 / x (where x is the number of white blood cells)

Step 5.4. Introduce variation in observed counts of red and white blood cells

- draw observed red cell count, redcounts1, from the Gamma distribution using shape parameter of $\text{redcount}/\phi$ and scale parameter of $\phi = s^2\text{redcount}$. For justification of this choice of shape and scale parameters, see Section A1.2.

- draw observed white cell count, whitecounts1, using shape parameter of $\text{whitecount}/\phi$ and scale parameter of $\phi = s^2\text{whitecount}$

- if either of redcounts1 or whitecounts1 exceed what would be equivalent to $3\text{x}10^6$ parasites/microlitre, redraw until it isn't.

Step 5.5. Calculate the observed parasite counts

- The observed parasite counts using the observed red and white blood cell counts are

pobs1 = round(redcount1)*hct*125.6*1000/rper,

pobs2 = round(whitecount1)*8000/wper

Step 5.6. Finalise observed parasite counts

- Set the observed parasite counts to be

$$\text{pobs} = \begin{cases} \text{pobs1} & \text{redcount1}>1 \\ \text{pobs2} & \text{otherwise} \end{cases}$$

A1.2. Parasite counts with quasi Poisson with over-dispersion

Red and white cell counts are generated from a quasi-Poisson distribution with over-dispersion [1], that is a Gamma distribution with mean $\mu=\alpha\beta$ and variance $\phi\mu=\alpha\beta^2$. The parameters ϕ and μ/ϕ are the shape and scale parameters, respectively, for the Gamma distribution.

From the models fitted to the real patient data, the variance of the residuals in the linear part of the parasite-time profiles is s^2. That is,

$$\mathrm{var}(\log(p)-\log(\hat{p}))=s^2$$

where p is the observed parasite counts and $\hat{p}$ is the fitted parasite values.

In the generation of simulated patient data, the value of the model fitted parasite count at time $t=t_0$ is $\hat{p}_{t_0}=P_0e^{-kt_0}$, which is a constant given the initial parasitaemia, the half-life and the value t_0. The value of the observed parasite count can be calculated from the observed red blood cell count using

$$p=\mathrm{redcount}*\mathrm{hct}*125.6*1000/\mathrm{rper}$$

where hct is the hematocrit and counting is done per rper red blood cells. It follows that

$$\mathrm{var}(\log(\mathrm{redcount}*\mathrm{hct}*125.6*1000/\mathrm{rper}))=\mathrm{var}(\log(\mathrm{redcount})+\log(\mathrm{hct}*125.6*1000/\mathrm{rper}))=s^2$$

Since hct and rper are constants:

$$\mathrm{var}(\log(\mathrm{redcount}))=s^2$$

Using the Delta method approximation of a variance function, namely,

$$\mathrm{var}(f(x))\approx[f'(\bar{x})]^2\,\mathrm{var}(x)$$

allows the approximation

$$\left(\frac{1}{\mu}\right)^2\mathrm{var}(\mathrm{redcount})\approx s^2$$

where μ is the mean of the redcount variable. By the Gamma distribution, the variance of redcount is $\phi\mu$. Hence:

$$\left(\frac{1}{\mu}\right)^2\phi\mu\approx s^2$$

which gives

$$\phi\approx s^2\mu$$

The mean of the redcount variable was taken to be the transformed parasite count from the model fit (either the linear model for profiles with no lag phase or the cubic model for profiles with a lag phase):

$$\mu=\hat{p}\frac{\mathrm{rper}}{\mathrm{hct}*125.6*1000}$$

[1] Ver Hoef, Jay M., and Peter L. Boveng. Quasi-Poisson vs. negative binomial regression: how should we model overdispersed count data? *Ecology* 88, no. 11 (2007): 2766-2772.
