# Supplementary material for: Optimal sampling designs for estimation of Plasmodium falciparum clearance rates in patients treated with artemisinin derivatives
Source: Malar J. 2013 Nov 13;12:411. doi: 10.1186/1475-2875-12-411 (PMC3842737; doi:10.1186/1475-2875-12-411)
Supplement: Additional file 2 — Time-points for sampling schedules investigated in simulation study. [file 1475-2875-12-411-S2.pdf]

Additional file A2. Time-points for sampling schedules investigated in simulation study

Table A2.1 The time-points (in hours) included in each of the sampling schedules investigated in the simulation study

**Alternative sampling schedules**	**0**	**1**	**6**	**7**	**8**	**10**	**12**	**13**	**16**	**18**	**24**	**25**	**and then counting every …**						
													**6h until negative**	**6h to 48hr**	**8h until negative**	**12h until negative**	**12h to 48hr**	**24h until negative**	**24 h + next hour**
**S1**	X		X				X			X	X		X						
**S2**	X				X				X		X				X				
**S3**	X						X				X					X			
**S4**	X										X							X	
**S1a**	X		X				X			X	X			X		X			
**S1b**	X		X				X			X	X			X				X	
**S1c**	X		X				X			X	X			X					
**S1_24**	X		X				X			X	X					X			
**S2_24**	X				X				X		X					X			
**B1**	X		X				X				X					X			
**M1**	X	X									X	X							X
**M2**			X	X							X	X							X
**M3**							X	X			X	X							X
**O1**	X		X			X					X						X		
**O1a**	X		X			X					X					X			
**O1b**	X		X			X					X						X	X	
