# Supplementary material for: Optimal sampling designs for estimation of Plasmodium falciparum clearance rates in patients treated with artemisinin derivatives
Source: Malar J. 2013 Nov 13;12:411. doi: 10.1186/1475-2875-12-411 (PMC3842737; doi:10.1186/1475-2875-12-411)
Supplement: Additional file 3 — Additional results. [file 1475-2875-12-411-S3.pdf]

Additional file A3. Additional results

Table A3.1 The proportion of profiles (number/total) for which the alternative schedule AX (X = 1, 2, 3, 4) overestimated the reference HL. The second and third columns compare overestimation for the case when the lag phase (tlag) under the reference and alternative schedule AX were equal to the case when the tlag under the reference was nonzero but the tlag under the alternative schedule AX was zero. The third and fourth columns compare overestimation for the case when tobit regression was used for both the reference and alternative schedule AX to the case when the tobit regression was used under the alternative schedule AX, but not the reference schedule

Schedule	tlag under reference and alternative schedule AX equal	tlag under reference nonzero and tlag under alternative schedule AX zero	Tobit under reference and alternative schedule AX	Tobit under alternative schedule AX, but not reference
A1	51% (1127/2202)	95% (226/238)	42% (865/2052)	74% (498/670)
A2	57% (1258/2188)	98% (385/394)	55% (1145/2067)	76% (501/656)
A3	61% (1222/2017)	97% (401/412)	54% (1127/2068)	77% (507/655)
A4	64% (1262/1983)	97% (542/557)	62% (1268/2049)	80% (541/673)

Table A3.2 The proportion of profiles for which there was a discrepancy between the HL from the reference and A4 schedules. Three indicator variables were considered: a lag phase under the reference schedule (non-zero versus zero), sufficient observations for a tobit regression in A4 schedule (yes versus no) and a six-hour delay in the time when the first negative parasitaemia was recorded when using A4 compared to the reference schedule (yes versus no).

Measure of discrepancy between the reference and A4 schedules	Lag phase under reference schedule		Sufficient observations for a tobit regression in A4 schedule		Six-hour delay in first negative (A4 versus reference)	
	Nonzero (N=711)	Zero (N=1,944)	No (N=226)	Yes (N=2,429)	Yes (N=856)	No (N=1,799)
Absolute difference in HL more than 0.5 hours	47%	19%	57%	24%	23%	28%
Relative difference in HL more than 10% in absolute value	73%	28%	87%	36%	36%	42%

Table A3.3 Performance summary of the simulation study, in which results from 16 different sampling schedules (Table 3) are presented, pooled across HL (two, three, four, five and six) and P0 (5,000, 10,000, 50,000, 100,000 and 200,000) values. That is, for each schedule 25,000 profiles were used to create the summary statistics (1,000 from each combination of HL and P0). For each schedule, “simulated” and “true” HLs were compared and the proportion of profiles with AD>1 hour, ARD>10%, ARD>20% and ARD>30% were calculated. The median, 98% range, 80% range and 50% range (IQR) are shown for both difference and relative difference distributions. The proportions of profiles that are misclassified when HLs are dichotomized using cutoff values of three, four, five and six hours are also shown. AD = absolute value of difference, ARD= absolute value of relative difference, IQR = interquartile range, RD = relative difference; prop = proportion.

Schedule	AD>1 hour	ARD>10%	ARD>20%	ARD>30%	Difference				Relative difference (RD)				Prop (%) profiles misclassified, with HL cutoff of:			
					Median	98% range	80% range	IQR	Median	98% range	80% range	IQR	3 h	4 h	5 h	6 h
S1	10	43	16	6	0	(-1.5, 1.9)	(-0.7, 0.7)	(-0.4, 0.3)	-1	(-36, 46)	(-18, 18)	(-9, 7)	0	0	0	0
S2	11	46	17	7	0	(-1.7, 2.0)	(-0.7, 0.7)	(-0.4, 0.3)	-1	(-37, 48)	(-18, 19)	(-10, 8)	9	10	11	8.5
S3	15	52	26	13	0.1	(-1.8, 2.2)	(-0.7, 1)	(-0.3, 0.5)	2	(-35, 70)	(-17, 31)	(-8, 15)	9	8.4	8.6	6.3
S4	44	66	49	38	0.9	(-1.2, 4.3)	(-0.4, 2.5)	(0.2, 1.8)	23	(-21, 161)	(-8, 93)	(3, 56)	23	28	23	14
M1	21	53	31	19	0.2	(-1.3, 3.4)	(-0.5, 1.5)	(-0.2, 0.8)	6	(-27, 87)	(-12, 45)	(-4, 24)	12	13	13	11
M2	13	43	19	9	0.1	(-1.5, 2.6)	(-0.7, 1.1)	(-0.3, 0.5)	2	(-33, 62)	(-16, 27)	(-7, 13)	8.8	10	11	9.4
M3	15	41	21	11	-0.1	(-3.4, 2.5)	(-1.1, 0.9)	(-0.6, 0.3)	-4	(-87, 58)	(-29, 21)	(-15, 8)	8.6	10	10	8.4
B1	13	48	20	8	0	(-1.8, 2.2)	(-0.8, 0.8)	(-0.4, 0.3)	-1	(-40, 54)	(-19, 21)	(-10, 9)	6	7	7.5	5.5
O1	15	49	22	10	-0.1	(-2.2, 2.4)	(-1.0, 0.8)	(-0.5, 0.3)	-2	(-44, 59)	(-23, 21)	(-13, 9)	7.5	9.7	11	7.1
O1a	13	47	20	8	0	(-1.8, 2.2)	(-0.7, 0.9)	(-0.4, 0.4)	-1	(-40, 55)	(-19, 22)	(-10, 10)	7	8	8.4	6.4
O1b	15	49	22	10	0	(-1.8, 2.6)	(-0.8, 0.9)	(-0.4, 0.4)	0	(-41, 58)	(-20, 24)	(-10, 10)	7.1	8.4	9.3	7.5
S1a	11	44	17	6	0	(-1.6, 2.0)	(-0.7, 0.7)	(-0.4, 0.3)	-1	(-37, 47)	(-18, 19)	(-10, 8)	0.2	1.4	3.6	3.4
S1b	12	47	19	7	0	(-1.6, 2.4)	(-0.7, 0.8)	(-0.4, 0.3)	-1	(-37, 52)	(-18, 21)	(-10, 9)	0.3	1.8	4.7	4.9
S1c	12	46	19	7	-0.1	(-1.9, 2.2)	(-0.9, 0.7)	(-0.5, 0.2)	-3	(-39, 53)	(-21, 18)	(-12, 7)	0.4	2.7	5.8	4.5
S1_24	12	46	20	8	0	(-1.7, 2.1)	(-0.8, 0.8)	(-0.4, 0.3)	-1	(-39, 52)	(-19, 21)	(-10, 9)	4.8	6.3	6.9	5
S2_24	13	48	19	8	0	(-1.8, 2.2)	(-0.8, 0.8)	(-0.4, 0.3)	-1	(-39, 53)	(-18, 21)	(-10, 9)	8.1	8.8	9.5	7.1
